# It’s Harder to Break a Relationship When you Commit Long

**DOI:** 10.1371/journal.pone.0156482

**Published:** 2016-06-06

**Authors:** Manabu Arai, Chie Nakamura

**Affiliations:** 1 Department of Economics, Seijo University, Tokyo, Japan; 2 Department of Linguistics and Philosophy, Massachusetts Institute of Technology, Cambridge, Massachusetts, United States of America; 3 Department of Language and Information Sciences, The University of Tokyo, Tokyo, Japan; 4 Japan Society for the Promotion of Science, Tokyo, Japan; University of Akron, UNITED STATES

## Abstract

Past research has produced evidence that parsing commitments strengthen over the processing of additional linguistic elements that are consistent with the commitments and undoing strong commitments takes more time than undoing weak commitments. It remains unclear, however, whether this so-called *digging-in* effect is exclusively due to the length of an ambiguous region or at least partly to the extra cost of processing these additional phrases. The current study addressed this issue by testing Japanese relative clause structure, where lexical content and sentence meaning were controlled for. The results showed evidence for a digging-in effect reflecting the strengthened commitment to an incorrect analysis caused by the processing of additional adjuncts. Our study provides strong support for the dynamical, self-organizing models of sentence processing but poses a problem for other models including serial two-stage models as well as frequency-based probabilistic models such as the surprisal theory.

## Introduction

In the processing of temporarily ambiguous sentences, comprehenders tend to experience greater processing difficulty at disambiguating input following a long ambiguous region with additional adjuncts than following a short region without such adjuncts. This is called a length effect or a *digging-in* effect and has been documented in many studies (e.g., [1–4]). Although various accounts have been proposed in an attempt to explain this phenomenon, it is difficult to distinguish between those accounts based on the results from previous studies because their experiments failed to control for the number and content of lexical items. The current study addresses this issue by manipulating the word order with adjuncts while controlling for the confounds.

The idea that comprehenders immediately build a tentative structure based on any available cues in input is consistent with processing models such as lexicalist constraint-based models as well as the more recent surprisal theory [5–7]. These models assume word-by-word incremental processing of incoming input; at each word comprehenders estimate and update the probabilities for possible syntactic analyses based on the input received so far. The surprisal theory further posits that the processing cost of new input is correlated with the size of a change in the probability distribution (i.e., *surprisal*) from previous input to the current input [7]. In processing a temporarily ambiguous sentence, there are multiple structural options available before ambiguity is resolved. An analysis would be assigned high probability when it is supported by the input received prior to disambiguating information. However, if the disambiguating input is incompatible with the probable analysis and supports an analysis that was relatively unexpected, the discrepancy causes a large change in the probability distribution and incurs great processing difficulty, which is known as a garden-path effect.

For example, in processing a sentence (1), comprehenders tend to analyze the noun phrase *the town* as a direct object of the initial verb *invaded* because the transitive structure occurs more frequently than the intransitive structure in English and also because the verb *invade* is more frequently used in the former structure than in the latter structure (cf. [5]).

(1) After the Martians invaded the town that the city bordered was evacuated. [1]. Importantly, it was shown that comprehenders commit more strongly to the direct object analysis by processing the following relative clause modifier *that the city bordered*. As a consequence, comprehenders experience greater processing cost at the disambiguating information (*was evacuated*) following the lengthening adjunct phrase compared to when the sentence does not contain such a phrase.

[1] found that people judged garden-path sentences to be less grammatical in a grammaticality judgment task when the ambiguous region was lengthened with an embedded relative clause (RC) following the postverbal NP as in (1) compared to the same sentences without the modifier (i.e., *After the Martians invaded the town was evacuated*), with the former judged grammatical 51% less often than the latter (in Experiment 1). They also found a similar effect of ambiguous phrase length for sentences such as (2a) and (2b). Assuming that a RC in (2a) is syntactically more complex than a prepositional phrase in (2b) as the former contains a greater number of syntactic nodes than the latter, this finding indicates that the length effect neither originates from nor is modulated by syntactic complexity per se.

(2a) When the men hunt the birds that cheetahs eat typically scatter. (2b) When the men hunt the birds with bright plumage typically scatter. Importantly, Ferreira and Henderson did not observe a comparable effect when the noun phrase was lengthened with additional prenominal adjectives (e.g., *the small and friendly town*). They suggested that the parser assigns thematic roles to noun phrases as soon as phrasal heads are encountered and claimed that this length effect is due to the distance between the head of an ambiguous phrase and the disambiguating word. The authors replicated this finding with a cumulative region-by-region self-paced reading task (See also [8]).

[3] replicated the finding of [1] using a gerund as the lengthener as in (3) with a self-paced reading task. They found longer reading times at the sentence-final disambiguating word (*grew*) for the sentences with the gerund phrase *describing Babylon* than those without it. No length effect was observed with the late-closure counterpart of these sentences (e.g., *As the author wrote the essay the book* (*describing Babylon*) *grew*).

(3) As the author wrote the book describing Babylon grew. Tabor and Hutchins argue that their results are best explained by dynamical, self-organizing models [9]. The models account for the digging-in phenomenon in terms of continuous interactions among small structural elements resulting in a self-organized, group-level structure that is maximally consistent with available constraints. In the above example, when the noun *the book* is perceived, its tree fragment becomes activated. The fragment forms a link with a node of the other fragment activated by the verb *wrote* because the verb takes a direct object and these fragments are adjacent, forming a *late closure* parse. Crucially, the strength of the link grows stronger over time through bidirectional interactions (called *inter-link feedback*) among all the linked nodes. Since the additional adjunct *describing Babylon* is consistent with the activated late closure analysis syntactically as well as lexico-semantically, processing this adjunct phrase further boosts the activation of the analysis. The strengthened activation of the incorrect analysis results in greater processing cost on encountering the sentence-final verb *grew* because it requires the parser to undo the incorrect analysis and build the correct intransitive (*early closure*) analysis. This temporal dynamics of self-organization of structural nodes is formally implemented in their parser called SOPARSE, which makes an explicit prediction about the digging-in effect.

One concern about these findings is that the contrasted sentences in previous studies differed not only in the length of ambiguous input but also in lexical content; the sentences with a long ambiguous region contains a greater number of lexical items than those with a short ambiguous region. The former sentences can incur greater processing cost simply due to the extra memory load or richer contextual information added by these additional words, rather than due to the length of ambiguous input itself. Furthermore, previous studies demonstrated that additional lexical items do not always lead to greater cost. In fact, [8] reported shorter reading times for sentences with a long ambiguous phrase, suggesting that comprehenders speed up in reading as the number of words increases. This trend is compatible with an anti-locality effect predicted by the surprisal or expectation-based theory (cf. [10]). This implies that the materials that contained extra lengthening phrases in previous studies may have incurred two contradictory effects; the extra phrases cause both a speed-up due to greater constraints on following input and a slow-down at disambiguating input due to stronger commitment to an incorrect analysis (cf. [11]). Therefore, it is difficult to determine from previous studies the extent of the influence that the distance between an ambiguous head and the disambiguating word has, independently of the slow-down due to the greater number of lexical items. The current study therefore examines the structure that has similar ambiguity but allows us to control for both the number of words as well as their content.

Using an eye-tracking reading technique, we investigated the processing of Japanese RC sentences such as (4).

(4) *Akachan-ga miruku-o koboshita joyuu-o mitsumeta*. Baby-NOM [milk-ACC spilled] actress-ACC stared at. ‘The baby stared at the actress who spilled the milk.’

In Japanese, a RC modifier (*miruku-o koboshita* 'spilled milk' in this example) is prenominal; it precedes the lexical head (*joyuu*, 'actress') without an overt complementizer or any grammatical marking on the verb, which makes the initial part of the sentence up to the RC verb identical with a single clause structure, causing temporary ambiguity. Many studies have shown that Japanese comprehenders incrementally process and associate noun phrases (NP) before a verb is processed [12–16]. Thus, they initially analyze the verb phrase as a part of a single clause with *the baby* as subject and *the milk* as a direct object and later experience processing difficulty at the RC head that forces them to revise the single clause interpretation for the correct embedded RC structure. Importantly for the current purpose, adjunct phrases that modify the RC verb phrase can be inserted either before the direct object in the RC or following it. We used the type of adverbial phrases called *verb phrase adverbs* (see [17] for details). [18] used behavioural data and [19] used corpus analysis to show that the canonical position of the verb phrase adverbs that denote the manner of an action (e.g., *yukkuri*, 'slowly') is both before and after the direct object NP, while it is unnatural to appear before the subject NP. These studies show that Japanese RC sentences are equally grammatical both when adverbial adjuncts appear before the RC direct object and when they appear following it. This is crucial because the difference in processing difficulty at the disambiguating region in our material, if observed, cannot be due to any difference in grammaticality or naturalness between the two word orders. Assuming that these adjuncts themselves would not prompt building of a MC or RC structure (none of existing models that we know of predict this), this allows us to manipulate how long readers retain their commitment to the incorrect syntactic analysis before the ambiguity is resolved while controlling for lexical content. This way, any difference between the two word orders must reflect an effect due to the duration for maintaining the incorrect analysis.

In addition to the linear order of the RC direct object and adjunct phrases, we also manipulated plausibility of the two analyses (MC and RC) by varying the RC direct object noun. As with many studies in English that demonstrated an influence of plausibility on the processing of structural ambiguities [20–22], [15] demonstrated that plausibility of the MC analysis influenced the comprehenders’ commitment to the analysis and the cost for revision in processing Japanese RC sentences. Like their study, we manipulated the plausibility of subject-direct object associations. In the MC-plausible condition, readers saw a RC direct object that was semantically highly related with the subject noun (milk). Readers are likely to associate it with the subject noun quickly and as a result commit strongly to a single clause analysis. In the RC-plausible condition, in contrast, they saw a RC direct object that was not related with the subject noun (champagne). In this condition readers are expected to make a weak commitment to a single clause analysis due to the weak association between the two noun phrases. Importantly, the verb was kept constant across conditions and would not affect the commitment (predicating the verb phrase with the subject noun is perfectly grammatical in all the conditions; for example, it is utterly possible for a baby to knock over and spill a bottle of champagne). The effect of plausibility would be reflected by the processing difficulty at the RC head. In the MC-plausible condition, we predict that readers would experience large cost for suppressing the strongly committed initial analysis and building the correct RC analysis. In contrast, readers are expected to experience smaller processing cost at the RC head because of the weaker commitment to the MC analysis with the RC-plausible NP. The manipulation of Plausibility was crossed with Word Order, resulting in the following four conditions (5).

(5a) Direct Object-Adjuncts order + MC-plausible. *Akachan-ga | miruku-o teeburu-de hadeni koboshita | joyuu-o | jitto | mitsumeta*. Baby-NOM |[milk-ACC on the table wildly spilled] | actress-ACC | fixedly | stared at. ‘The baby fixedly stared at the actress who spilled the milk wildly on the table.’

(5b) Direct Object-Adjuncts order + RC-plausible. *Akachan-ga | shanpan-o teeburu-de hadeni koboshita | joyuu-o | jitto | mitsumeta*. Baby-NOM |[champagne-ACC on the table wildly spilled] | actress-ACC | fixedly | stared at. ‘The baby stared fixedly at the actress who spilled the champagne wildly on the table.’

(5c) Adjuncts- Direct Object order + MC-plausible. *Akachan-ga | teeburu-de hadeni miruku-o koboshita | joyuu-o | jitto | mitsumeta*. Baby-NOM | [on the table wildly milk-ACC spilled] | actress-ACC | fixedly | stared at.

(5d) Adjuncts- Direct Object order + RC-plausible. *Akachan-ga | teeburu-de hadeni shanpan-o koboshita | joyuu-o | jitto | mitsumeta*. Baby-NOM | [on the table wildly champagne-ACC spilled] | actress-ACC | fixedly | stared at.

To confirm the strong preference for the MC analysis in Japanese, we conducted a sentence completion norming study. In this study, participants were asked to produce the continuation of the sentence fragment excluding the initial verb of our experimental items onward (i.e., *Akachan-ga miruku -o teeburu-de hadeni……* ‘Baby-NOM milk-ACC on the table wildly……’). We created 4 lists in a Latin-square design with 24 experimental items. Each list included 96 fillers and was presented in pseudo-random order. The study was conducted over the internet using a Japanese crowdsourcing service *Lancers* (http://www.lancers.jp/). The survey started with an instruction page and two practice trials. Two hundred and eight participants in total took part in this survey. The completed sentences were manually coded either as MC or RC. The results are summarized in Table 1.

**Table 1:** The Number of MC, RC, and Ungrammatical Completions Produced per Condition in the Sentence Completion Test.

	Main clause	Relative clause	Ungrammatical
Direct Object-Adjuncts order + MC-plausible	1239	1	8
Direct Object-Adjuncts order + RC-plausible	1237	6	5
Adjuncts- Direct Object order + MC-plausible	1247	1	0
Adjuncts- Direct Object order + RC-plausible	1236	11	1

There were 14 invalid responses. Among these, 13 ungrammatical completions occurred in the Direct Object-Adjuncts order condition and contained another accusative NP presumably because participants forgot about the previously encountered direct object due to the intervening adjunct phrases. The other erroneous response indicated that a participant failed to understand a direct object noun in an item. The results confirmed a very strong preference for the MC over the RC structure across all the conditions. Importantly, they showed no evidence that the MC structure is more likely to follow in the Direct Object-Adjuncts order than in the Adjuncts-Direct Object order when the direct object was plausible for the MC structure and demonstrate that the position of the adjunct phrases does not affect the preference for the MC or RC structure. To further confirm this, we also checked the frequency of the RC structure following the roughly equivalent initial ambiguous part of the sentences in alternating word orders with the Balanced Corpus of Contemporary Written Japanese (BCCWJ) using a web-based interface called *Chunagon* (http://pj.ninjal.ac.jp/corpus_center/bccwj/). There were 406 instances in the Adjuncts-Direct Object order and 180 instances in the Direct Object-Adjuncts order but none of the instances contained the RC structure, again demonstrating no influence of word order on the preference for the MC or RC structure.

In the current study, we predict that the strength of their commitment to the incorrect analysis would differ depending on our manipulations of Word Order and Plausibility. We assume that the amount of difficulty at the head would reflect how much comprehenders commit to the MC analysis prior to the disambiguating information. Here, we lay out our precise predictions concerning the sentences in each condition according to the dynamical self-organizing system. When readers see the RC direct object that is plausible for the MC analysis as in (5a, *miruku*, ‘milk’), they would immediately build a verb phrase and commit to the MC analysis strongly. The MC analysis should become more activated by processing the following adjunct phrases because they are consistent with the analysis, resulting in large processing cost at the disambiguating RC head (*joyuu*, ‘actress’). By contrast, when the adjunct phrases appeared before the RC direct object as in (5c), the activation of the verb phrase should be lower than in (5a) because readers would not build any structure on encountering the adjunct phrases and only start to build the MC verb phrase on encountering the following accusative noun. This should result in smaller processing cost at the RC head in (5c) than (5a). On the other hand, when readers encounter the RC direct object that is semantically plausible for the RC analysis as in (5b, *shanpan*, ‘champagne’), they would not build a verb phrase or commit to the MC analysis due to the implausibility of the analysis. Therefore, it would be relatively easy for readers to construct the correct RC structure following the RC head, predicting little processing difficulty. This would hold true whether the adjunct phrases appear before the accusative noun or after it, that is, no difference in processing cost is predicted between (5b) and (5d). In short, we predict an interaction between an effect of plausibility of the RC direct object and that of the position of the adjunct phrase at the disambiguating RC head.

The current study made use of eye-tracking technique in reading, which has a great advantage in investigating real-time dynamics of parsing processes as the data reflects non-segmented natural reading of experimental stimuli unlike a grammaticality judgment task or self-paced reading technique [1,8]. Also, lexical content as well as sentence meaning are controlled for in the current study, excluding confounding factors that existed in previous studies. This ensures that the predicted effect, if observed, occurs in normal and real-time sentence comprehension and must be taken as evidence for a digging-in effect exclusively due to the delay of disambiguating information.

## Experiment

### Subjects

Forty native speakers of Japanese with normal or corrected-to-normal vision were recruited from the student community at the University of Tokyo.

### Ethics Statement

We did not seek approval by an institutional review board for this study because it is not required to conduct a study of the type reported in this manuscript. It required all studies to comply with the Code of Conduct for Scientific Research at The University of Tokyo (http://www.u-tokyo.ac.jp/content/400030733.pdf) and we confirm that our study did. Submitted is a letter from The Committee on Ethics of Experimental Research on Human Subjects that certifies that ethical approval is not required for this study. All subjects gave written consent by signing a consent form before starting an experimental session. Their voluntary participation was compensated with small remuneration.

### Materials and Design

Twenty-four sets of experimental items were created with a 2 × 2 design (Word Order × Plausibility). We adopted the material from [15], which contains additional adjunct phrases following the RC object. We created the items in the Adjunct-RC object condition by moving the adjunct phrases before the RC object. Four lists of experimental materials were then prepared following a Latin square design. Each list included 60 fillers in addition to the experimental items and was presented in pseudo-random order (at least one filler sentence always intervened between experimental items). Each list also included 46 comprehension questions following filler sentences to make sure that participants comprehended the sentences. Every experimental session always started with 4 practice sentences along with two comprehension questions. All the experimental items used in the current study are provided in S1 Appendix.

### Procedure

We recorded participants' eye-movements while reading experimental items using an EyeLink II head-mounted eye-tracking system (SR-Research) at the sampling rate of 500 Hz. After brief instructions were provided, participants sat in front of a computer monitor and went through a brief calibration procedure. Before each trial, a small square appeared at the left edge of the screen in a vertically central position. The stimulus sentence was automatically presented on the screen following a brief gaze at the square. The location of the square corresponded to the position of the first letter of the sentence, ensuring that participants started to read from the leftmost word of the sentence. When participants finished reading, they pressed a button to move onto a next trial. Each experimental session took approximately 30 minutes.

### Data Analysis and Results

The average correct response rate to comprehension questions was 93.9% (SD = 4.6) and none of the participants were excluded from the analysis. In the analysis of fixation data, we first removed fixations that were either extremely long (> 1200 ms) or extremely short (< 80 ms) unless there was another fixation within one character distance from the short fixation, in which case the short fixation was integrated into the adjacent fixation [23]. Among various measures available, we report the results from three eye-movement measures in the current paper; *first-pass*, *right-bounded*, and *second-pass* reading times. First pass time is the sum of durations of the fixations in a particular region following the first entry in the region until the first fixation outside the region (either to the left or the right). Right-bounded reading time is the sum of the fixations in the region following the first entry in the region until the first fixation outside the region to the right. These two measures reflect an early process (i.e., before seeing any information following the region of interest) and we expect to observe a predicted digging-in effect in these measures as it reflects processing difficulty resulting from the pre-activation of the incorrect MC analysis. Second-pass reading time is the sum of fixations made in a region after the region has already been exited to the right. The second pass measure also included trials where the region was skipped (i.e., zero reading time), while the other two measures excluded those trials. Second-pass time is often referred to as a late measure, potentially reflecting the cost for forming the correct structural relationship or/and undoing the initial incorrect analysis [24]. We excluded the reading times that were either less or greater than 3 standard deviations away from the average reading time. Each sentence was divided into regions (as indicated by | in (5)) for the analysis of fixation data. In the following section, we report the results from the critical disambiguating RC head region (*joyuu-o*, ‘actress’), where an ambiguity effect is expected to occur. We also report the results from the pre-critical region (*koboshita*, *‘spilled’*), which may reflect an effect due to parafoveal previewing.

We analyzed the reading times of the above three measures for these regions using Linear Mixed-Effects (LME) models [25], entering Word Order (Direct Object-Adjuncts or Adjuncts-Direct Object) and Plausibility (MC-plausible vs. RC-plausible), as well as the interaction between the two factors as fixed effects, and participants and items as random effects. All the fixed effects are centered to have a mean of 0 and a range of 1 to minimize collinearity. The initial model also included random slopes for the fixed effects of both participant and item random effects. We explored the best-fit model for each analysis using a backward selection approach and report the results from the optimal model below. We report coefficients (*β*), standard errors (*SE*), *t*-values, and their *p*-values from the best-fit model. We computed the *p*-value for each factor using likelihood ratio (LR) tests. The pairwise comparisons for simple effects are conducted using a 95% confidence interval, which is estimated as 2 SE of the coefficient of Word Order in the best-fit model. Table 2 shows the mean reading times for the three eye-movement measures in the critical region for each condition.

**Table 2:** Mean Reading Times (and Standard Errors) in Milliseconds for First Pass, Right-bounded, and Second Pass Measures in the Critical Region.

	First pass	Right-bounded	Second pass
Direct Object-Adjuncts + MC-plausible	293 (14)	342 (18)	245 (22)
Direct Object-Adjuncts + RC-plausible	274 (15)	304 (15)	202 (23)
Adjuncts- Direct Object + MC-plausible	280 (11)	316 (16)	200 (24)
Adjuncts- Direct Object + RC-plausible	287 (13)	319 (16)	172 (23)

**Fig 1:**
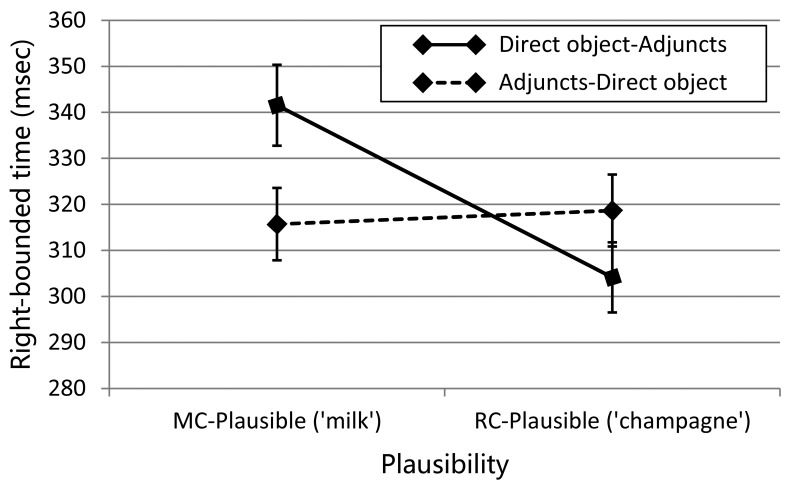
Right-bounded times in the critical region. Error bars show by-participants standard errors.

The analysis of first pass reading times showed neither a main effect of Word Order (*p* = 0.754) nor that of Plausibility (*p* = 0.374). There was a trend for an interaction between the two factors (β = 28.98, SE = 18.00, *t* = 1.61, *p* = 0.107). This non-significant trend may be due to the existence of the short fixations that occurred before launching regressive eye-movements to earlier regions as a result of processing difficulty (e.g., [15, 26]). We therefore examined the first pass times only for the trials in which no such regressive eye-movements occurred (called *first pass with no regression* times). The analysis revealed a significant interaction between Plausibility and Word Order (β = 41.57, SE = 20.73, *t* = -2.01, *p*<0.05). Planned comparisons showed that the simple effect of Word Order was reliable when the RC object was plausible for the MC analysis (23 ms; CI = 21 ms), showing that the disambiguating RC head following the MC-plausible direct object was read more slowly when the direct object appeared before the adjuncts than when it appeared after the adjuncts. On the other hand, the contrast was not reliable when the RC object was implausible (19 ms). In order to estimate the effect size for the interaction, we calculated *partial η*^*2*^, which is defined as effect variance in proportion to effect variance plus corresponding error variance (SSeffect/(SSeffect + SSerror)) [27]. We report the averages across participant and item analyses. The analysis revealed a *partial η*^*2*^ of 0.068 for the interaction, which falls within the range of "a medium effect" (between 0.06 and 0.139). We also conducted power analysis with the obtained *partial η*^*2*^ value (at alpha = 0.05) using a statistical power analysis program G*Power [28]. The power for the interaction is 0.816 for the sample size of this study (i.e., 40). The analysis of right-bounded times showed neither a main effect of Word Order (*p* = 0.460) nor an effect of Plausibility (*p* = 0.249), but there was a significant interaction between the two factors (β = 46.46, SE = 23.22, *t* = -2.00, *p*<0.05). Again, the simple effect of Word Order was reliable when the RC object was plausible for the MC analysis (26 ms; CI = 23 ms) but not reliable when it was plausible for the RC analysis (15 ms). Fig 1 illustrates the pattern of the interaction (The original data of the right-bounded times in the critical region are provided in S2 Appendix). This interaction has a medium effect size (*partial η*^*2*^ = 0.123) and the observed power for the sample size of 40 is 0.98, which is fairly robust (the required sample size with the power at alpha = 0.05 is 32).

The analysis of second pass times showed a main effect of Word Order (β = -39.30, SE = 15.43, *t* = -2.55, *p*<0.05). Participants spent more time in re-reading this region when the adjunct phrase was positioned after the RC object than when it was positioned before, suggesting that it was harder to form and interpret the correct RC structure following a lengthened ambiguous region than following a short region. The analysis also showed a main effect of Plausibility (β = -35.08, SE = 15.44, *t* = -2.27, *p*<0.05). Participants spent more time in re-reading this region in the MC-plausible condition than in the RC-Plausible condition, reflecting a greater processing cost when the RC object was thematically appropriate for the incorrect MC analysis than when it was not. No interaction was observed with this measure. (*p* = 0.586).

Next, we analyzed the reading times in the pre-critical region (*koboshita*, ‘spilled’), which reflects a possible effect of parafoveal previewing (see [29] for an effect in reading in Japanese; [30], for a general summary). The analysis showed the same pattern as in the critical region. The analysis of first pass with no regression times revealed a marginally significant interaction between the two factors (β = 31.95, SE = 17.52, *t* = 1.82, *p* = 0.069). The effect has a medium effect size (*partial η*^*2*^ = 0.082, observed power = 0.967). The analysis of right-bounded times in this pre-critical region also showed a marginally significant interaction with the same pattern (β = 35.05, SE = 18.91, *t* = 1.85, *p* = 0.064). The effect had a medium effect size (*partial η*^*2*^ = 0.089, observed power = 0.919). Analysis of second-pass times showed a marginally significant effect of Word Order (β = -30.09, SE = 16.26, *t* = -1.85, *p* = 0.064) and a significant effect of Plausibility (β = -68.85, SE = 16.27, *t* = -4.23, *p* < .001). There was no interaction between the two factors (*p* = 0.843). Therefore, the pattern of results in this pre-critical region is almost identical to that in the critical region, although the observed effects tend to be weaker in the pre-critical region.

Additionally, we conducted a combined analysis on the first pass with no regression times in the pre-critical and critical regions. This analysis was performed to test a difference in the interactions between Word Order and Plausibility in the two regions. Note that the first pass with no regression times in the two regions in any given trial are composed of different fixations. We entered Region (pre-critical or critical) as an additional fixed effect in the model, allowing an three-way interaction between Word Order, Plausibility, and Region as well as all the two-way interactions and their main effects. The analysis revealed no three-way interaction nor other effects but there was a significant two-way interaction between Word Order and Plausibility (β = 36.75, SE = 13.64, *t* = 2.70, *p* < 0.01), which had a medium effect size (*partial η*^*2*^ = 0.116, observed power = 0.978), demonstrating that the two regions showed the consistent pattern of the interaction between Word Order and Plausibility. The simple effect of Word Order was reliable when the RC direct object was plausible for the MC structure (27 ms; CI = 14 ms). This provides further support for a digging-in effect with a lengthened ambiguous region. Interestingly, the simple effect of Word Order was also reliable when the RC direct object was plausible for the RC structure (20 ms). This suggests that participants experienced less processing difficulty when the direct object appeared before the adjuncts than when it appeared after the adjuncts. This implies digging-in of the RC structure; since the direct object was highly plausible for the RC structure, readers activated the RC structure on encountering the RC object and its activation strengthened over the processing of following adjuncts.

To add, we also examined reading times in the post-critical region (*sotto*, ‘with care’). The analysis of any measures showed no interaction between Word Order and Plausibility (*p*s > .10), demonstrating that there was no spill-over effect.

## General Discussion

The current study investigated the influence of additional adjuncts on the cost of structural ambiguity, i.e., a digging-in effect, with Japanese RC sentences. Unlike previous studies, our study manipulated only the linear distance between the RC object and its head by inserting extra adjunct phrases either before or after the RC object, while controlling the number of lexical items. We also manipulated the plausibility of the MC or RC analyses with the RC direct object.

Our results showed that participants experienced greater processing difficulty at the disambiguating head when the RC direct object was thematically plausible for the MC analysis and appeared before additional adjuncts, as compared to when it was plausible but appeared following the adjuncts. The results demonstrated that participants formed the direct object analysis as soon as they encountered any RC direct object that was thematically plausible as a direct object of the initial verb, and then made a stronger commitment to the analysis when extra adjuncts were encountered. In contrast, we observed no influence of word order when the RC direct object was thematically implausible for the MC analysis, suggesting that the direct object analysis was not activated in this case.

Now we consider the implications of our results for sentence processing models. First, our results provide clear evidence against modular two-stage processing models such as the garden path theory because these models assume that syntactically non-mandatory adjunct phrases do not contribute to the complexity of syntactic structures and thus their position in a sentence would not influence structural analysis. The construal hypothesis, proposed by [31–32], allows underspecification and variable structural choices for the association of adjunct (called *nonprimary*) phrases. Unlike the garden path theory, the hypothesis allows non-structural information to influence the association with adjunct phrases. However, these adjunct phrases are optional and no lexical specifications exist between heads and the phrases, suggesting that the adjunct phrases themselves do not influence syntactic relations of primary phrases and the choice of association, even if it contradicts with the initial preference, does not incur processing cost. Therefore, the construal hypothesis also predicts no influence of the position of adjunct phrases.

These models also have difficulty in accounting for an immediate influence of plausibility because non-syntactic lexically-based information, such as plausibility, is assumed to have no influence on the selection of the initial analysis (e.g., [21]). Some other two-stage models such as the unrestricted race model allow unrestricted use of any source of information in determining the initial analysis but fail to explain our results [33–34]. The unrestricted race model can account for the main effect of Plausibility as it posits that different lexical items would result in different structural biases. However, it cannot account for the effect of word order or its interaction with plausibility as lexically identical sentences in different word orders were contrasted in the current study. Our results are also problematic for the models that assume strict head-driven parsing principles (e.g., [35]). These models assume that the lexical head licenses argument structures so that adjunct phrases should not influence structural processing. They also predict that the RC structure should not be projected before readers encountered the RC head, which was also contradicted by our results.

Furthermore, our results are not predicted by frequency-based probabilistic accounts such as the surprisal theory and expectation-based models. These accounts assume that the MC analysis is highly activated on encountering the RC direct object because the MC structure occurs highly frequently with an accusative argument following a nominative argument in Japanese. The MC structure would be assigned higher probability when the accusative argument was thematically plausible for the MC analysis than when it was not plausible. Crucially, the processing of following adjuncts would further increase the probability of the MC analysis if the MC structure is in fact more probable when the adjuncts appear after the RC direct object than when they appear before it. The results from our norming study as well as the corpus search failed to show any evidence for this; there was no difference in the frequency of the MC and RC structures between the sentences in the two word orders in our study. The results suggest that comprehenders expect the MC structure with equal probability before encountering the RC head regardless of whether adjunct phrases appear before the direct object or after it. Instead, the results are fully compatible with the dynamical, self-organizing parser. It predicts that the MC analysis is formed on encountering the RC direct object supporting the MC analysis and the commitment to the analysis strengthens on processing following adjuncts because the attachment of the adjunct phrases to the verb phrase forms a highly plausible interpretation of the MC analysis and the adjuncts allow more time for interactions between the nodes in the verb phrase parse, resulting in the strengthened commitment to the MC analysis. On the other hand, no verb phrase parse is built when adjuncts are encountered prior to the direct object. As a result, the presence of the adjuncts in this case hardly affects the verb phrase parse and leads to a weaker commitment to the MC analysis.

## Conclusions

The current study examined the effect of ambiguous phrase length in the comprehension of temporarily ambiguous Japanese relative clause sentences. Our results showed that when the relative clause direct object was plausible for the main clause analysis, our participants experienced greater ambiguity cost at the relative clause head when the adjuncts followed the direct object compared to when the adjuncts preceded it. This demonstrates that the incorrect main clause analysis was committed more strongly by processing additional adjuncts after incorrectly attaching the direct object to the subject noun phrase. The finding fits well with the dynamical self-organizing models, which predict that the strength of structural commitments changes dynamically over the time allowed by these additional adjuncts. In contrast, since lexical content was controlled for and there was no difference in the frequency of the relative clause structure for the different word orders tested in our study, our results pose a problem for serial modular models such as the garden path theory as well as for frequency-based probabilistic accounts such as the surprisal theory.

## Supporting Information

S1 Appendix Experimental items used in the current study. (DOCX)

S2 Appendix Right-bounded time data in the critical region along with the descriptions of variables. The data accessible to readers comply with the Code of Conduct for Scientific Research at The University of Tokyo, and do not reveal any kind of privacy about participants. The use of data is restricted to non-profit, scholarly research purposes only.
(CSV)
