# Supplementary material for: It’s Harder to Break a Relationship When you Commit Long
Source: PLoS One. 2016 Jun 6;11(6):e0156482. doi: 10.1371/journal.pone.0156482 (PMC4894567; doi:10.1371/journal.pone.0156482)
Supplement: S1 Appendix — (DOCX) [file pone.0156482.s001.docx]

Item number + Condition number + Sentences

Condition 1 = Direct Object-Adjuncts order + MC-plausible

Condition 2 = Direct Object-Adjuncts order + RC-plausible

Condition 3 = Adjuncts-Direct Object order + MC-plausible

Condition 4 = Adjuncts-Direct Object order + RC-plausible

1. 1. サラリーマンがネクタイを引き出しに大切そうに持っているOLにそっと話しかけた。

Businessman-NOM necktie-ACC in a drawer protectively had office lady quietly talked

‘The businessman talked quietly to the office lady who had a necktie protectively in a drawer.’

1. 2. サラリーマンが口紅を引き出しに大切そうに持っているOLにそっと話しかけた。

Businessman-NOM lipstick-ACC in a drawer protectively had office lady quietly talked

‘The businessman talked quietly to the office lady who had a lipstick protectively in a drawer.’

1. 3. サラリーマンが引き出しに大切そうにネクタイを持っているOLにそっと話しかけた。

Businessman-NOM in a drawer protectively necktie-ACC had office lady quietly talked

1. 4. サラリーマンが引き出しに大切そうに口紅を持っているOLにそっと話しかけた。

Businessman-NOM in a drawer protectively lipstick-ACC had office lady quietly talked

2. 1. 男性がバイクをお店でじっと見つめている女の子を大声で呼び止めた。

Man-NOM motorcycle-ACC at the score fixedly stared girl loudly called

‘The man called loudly the girl who was staring at the motorcycle fixedly at the store.’

2. 2. 男性がキャンディーをお店でじっと見つめている女の子を大声で呼び止めた。

Man-NOM candy-ACC at the store fixedly stared girl loudly called

‘The man called loudly the girl who was staring at the candy fixedly at the store.’

2. 3. 男性がお店でじっとバイクを見つめている女の子を大声で呼び止めた。

Man-NOM at the store fixedly motorcycle-ACC stared girl loudly called

2. 4. 男性がお店でじっとキャンディーを見つめている女の子を大声で呼び止めた。

Man-NOM at the store fixedly candy-ACC stared girl loudly called

3. 1. 高校生がサッカーを公園で楽しそうにしている老人にさりげなく近づいた。

High school student-NOM soccer-ACC in the park joyfully was doing old man casually approached

‘The high school student approached casually to the old man who was playing soccer joyfully in the park.’

3. 2. 高校生がゲートボールを公園で楽しそうにしている老人にさりげなく近づいた。

High school student-NOM gate ball-ACC in the park joyfully was doing old man casually approached

‘The high school student approached casually to the old man who was playing gate ball joyfully in the park.’

3. 3. 高校生が公園で楽しそうにサッカーをしている老人にさりげなく近づいた。

High school student-NOM in the park joyfully soccer-ACC was doing old man casually approached

3. 4. 高校生が公園で楽しそうにゲートボールをしている老人にさりげなく近づいた。

High school student-NOM in the park joyfully gate ball-ACC was doing old man casually approached

4. 1. 3歳児がおもちゃを手さげの中に大切そうに持っているビジネスマンに小さな声で話しかけた。

3-year-old kid-NOM toy-ACC in his bag protectively had businessman quietly talked

‘The 3-year-old kid talked quietly to the businessman who had a toy protectively in his bag.’

4. 2. 3歳児が缶ビールを手さげの中に大切そうに持っているビジネスマンに小さな声で話しかけた。

3-year-old kid-NOM beer can-ACC in his bag protectively had businessman quietly talked

‘The 3-year-old kid talked quietly to the businessman who had a beer can protectively in his bag.’

4. 3. 3歳児が手さげの中に大切そうにおもちゃを持っているビジネスマンに小さな声で話しかけた。

3-year-old kid-NOM in his bag protectively toy-ACC had businessman quietly talked

4. 4. 3歳児が手さげの中に大切そうに缶ビールを持っているビジネスマンに小さな声で話しかけた。

3-year-old kid-NOM in his bag protectively beer can-ACC had businessman quietly talked

5. 1. 赤ちゃんがミルクをテーブルで派手にこぼした女優をじっと見つめた。

Baby-NOM milk-ACC on the table wildly spilled actress fixedly stared

‘The baby stared fixedly at the actress who spilled milk wildly on the table.’

5. 2. 赤ちゃんがシャンパンをテーブルで派手にこぼした女優をじっと見つめた。

Baby-NOM champaign-ACC on the table wildly spilled actress fixedly stared

‘The baby stared fixedly at the actress who spilled champaign wildly on the table.’

5. 3. 赤ちゃんがテーブルで派手にミルクをこぼした女優をじっと見つめた。

Baby-NOM on the table wildly milk-ACC spilled actress fixedly stared

5. 4. 赤ちゃんがテーブルで派手にシャンパンをこぼした女優をじっと見つめた。

Baby-NOM on the table champaign-ACC wildly spilled actress fixedly stared

6. 1. ロックスターがギターを腕の中に大切そうに持っている小学生にそっと声をかけた。

Rock star-NOM guitar-ACC in his arms protectively had schoolchild quietly talked

‘The rock star talked quietly to the schoolchild who had a guitar protectively in his arms.’

6. 2. ロックスターがランドセルを腕の中に大切そうに持っている小学生にそっと声をかけた。

Rock star-NOM school bag-ACC in his arms protectively had schoolchild quietly talked

‘The rock star talked quietly to the schoolchild who had a school bag protectively in his arms.’

6. 3. ロックスターが腕の中に大切そうにギターを持っている小学生にそっと声をかけた。

Rock star-NOM in his arms protectively guitar-ACC had schoolchild quietly talked

6. 4. ロックスターが腕の中に大切そうにランドセルを持っている小学生にそっと声をかけた。

Rock star-NOM in his arms protectively school bag-ACC had schoolchild quietly talked

7. 1. 医師が白衣を鞄の中にこっそり持っている高校生をあわてて追いかけた。

Doctor-NOM white coat-ACC in his bag secretly had high school student in a hurry chased

‘The doctor chased the high school student who had a white coat secretly in his bag in a hurry.’

7. 2. 医師が学生服を鞄の中にこっそり持っている高校生をあわてて追いかけた。

Doctor-NOM school uniform-ACC in his bag secretly had high school student in a hurry chased

‘The doctor chased the high school student who had a school uniform secretly in his bag in a hurry.’

7. 3. 医師が鞄の中にこっそり白衣を持っている高校生をあわてて追いかけた。

Doctor-NOM in his bag secretly white coat-ACC had high school student in a hurry chased

7. 4. 医師が鞄の中にこっそり学生服を持っている高校生をあわてて追いかけた。

Doctor-NOM in his bag secretly school uniform-ACC had high school student in a hurry chased

8. 1. 小学生がマンガをお店で急いで買ってきた先生にこっそりと話しかけた。

Schoolchild-NOM comic book-ACC at a store in a hurry bought teacher secretly talked

‘The schoolchild talked secretly to the teacher who bought a comic book in a hurry at a store.’

8. 2. 小学生が万年筆をお店で急いで買ってきた先生にこっそりと話しかけた。

Schoolchild-NOM fountain pen-ACC at a store in a hurry bought teacher secretly talked

‘The school kid talked secretly to the teacher who bought a fountain pen in a hurry at a store.’

8. 3. 小学生がお店で急いでマンガを買ってきた先生にこっそりと話しかけた。

Schoolchild-NOM at a store in a hurry comic book-ACC bought teacher secretly talked

8. 4. 小学生がお店で急いで万年筆を買ってきた先生にこっそりと話しかけた。

Schoolchild-NOM at a store fountain pen-ACC in a hurry bought teacher secretly talked

9. 1. 社長がタキシードをデパートで楽しげに選んでいた女性を食事に誘った。

CEO-NOM tuxedo-ACC at the department store happily was choosing lady dinner invited

‘The CEO invited the lady who was choosing a tuxedo happily at the department store to dinner.’

9. 2. 社長がイヤリングをデパートで楽しげに選んでいた女性を食事に誘った。

CEO-NOM earrings-ACC at the department store happily was choosing lady dinner invited

‘The CEO invited the lady who was choosing earrings happily at the department store to dinner.’

9. 3. 社長がデパートで楽しげにタキシードを選んでいた女性を食事に誘った。

CEO-NOM at the department store happily tuxedo-ACC was choosing lady dinner invited

9. 4. 社長がデパートで楽しげにイヤリングを選んでいた女性を食事に誘った。

CEO-NOM at the department store happily earrings-ACC was choosing lady dinner invited

10. 1. 市長が会議を早朝から元気にしている小学生を楽しげに見つめた。

Mayor-NOM meeting-ACC early in the morning excitedly was doing schoolchild amusingly stared

‘The mayor stared amusingly at the schoolchild who was having a meeting excitedly early in the morning.’

10. 2. 市長がくもんを早朝から元気にしている小学生を楽しげに見つめた。

Mayor-NOM *kumon*-ACC early in the morning excitedly was doing schoolchild amusingly stared

‘The mayor stared amusingly at the schoolchild who was studying *kumon* excitedly early in the morning.’

10. 3. 市長が早朝から元気に会議をしている小学生を楽しげに見つめた。

Mayor-NOM early in the morning excitedly meeting-ACC was doing schoolchild amusingly stared

10. 4. 市長が早朝から元気にくもんをしている小学生を楽しげに見つめた。

Mayor-NOM early in the morning excitedly *kumon*-ACC was doing schoolchild amusingly stared

11. 1. 女子高生が携帯をリビングで夢中になって触っている赤ちゃんを楽しそうに世話した。

High school girl-NOM cell phone-ACC in a living room enthusiastically touching baby happily took care

‘The high school girl happily took care of the baby who was enthusiastically touching a cell phone in a living room.’

11. 2. 女子高生がおしゃぶりをリビングで夢中になって触っている赤ちゃんを楽しそうに世話した。

High school girl-NOM pacifier-ACC in a living room enthusiastically touching baby happily took care

‘The high school girl happily took care of the baby who was enthusiastically touching a pacifier in a living room.’

11. 3. 女子高生がリビングで夢中になって携帯を触っている赤ちゃんを楽しそうに世話した。

High school girl-NOM in a living room enthusiastically cell phone-ACC touching baby happily took care

11. 4. 女子高生がリビングで夢中になっておしゃぶりを触っている赤ちゃんを楽しそうに世話した。

High school girl-NOM in a living room enthusiastically pacifier-ACC touching baby happily took care

12. 1. 浪人生が参考書を鞄の中にこっそりと持っている看護婦に嬉しそうに話を聞いた。

Gap year student-NOM textbook-ACC in bag secretly had nurse happily heard a story

‘The gap year student happily heard a story from a nurse who secretly had a textbook in a bag.’

12. 2. 浪人生が注射器を鞄の中にこっそりと持っている看護婦に嬉しそうに話を聞いた。

Gap year student-NOM injector-ACC in bag secretly had nurse happily heard a story

‘The gap year student happily heard a story from a nurse who secretly had an injector in a bag.’

12. 3. 浪人生が鞄の中にこっそりと参考書を持っている看護婦に嬉しそうに話を聞いた。

Gap year student-NOM in bag secretly textbook-ACC had nurse happily heard a story

12. 4. 浪人生が鞄の中にこっそりと注射器を持っている看護婦に嬉しそうに話を聞いた。

Gap year student-NOM in bag secretly injector-ACC had nurse happily heard a story

13. 1. 俳優が台本を病院の待合室で読んでいた受験生と思いがけず意気投合した。

Actor-NOM script-ACC at a hospital in the waiting lounge was reading student unexpectedly clicked

‘The actor clicked unexpectedly with the student who was reading a script in the waiting lounge of a hospital.’

13. 2. 俳優が教科書を病院の待合室で読んでいた受験生と思いがけず意気投合した。

Actor-NOM textbook-ACC at a hospital in the waiting lounge was reading student unexpectedly clicked

‘The actor clicked unexpectedly with the student who was reading a textbook in the waiting lounge of a hospital.’

13. 3. 俳優が病院の待合室で台本を読んでいた受験生と思いがけず意気投合した。

Actor-NOM at a hospital in the waiting lounge script-ACC was reading student unexpectedly clicked

13. 4. 俳優が病院の待合室で教科書を読んでいた受験生と思いがけず意気投合した。

Actor-NOM at a hospital in the waiting lounge textbook-ACC was reading student unexpectedly clicked

14. 1. 女の子がリコーダーを近所のレストランで落とした男性に力いっぱい手をふった。

Girl-NOM recorder-ACC neighboring at a restaurant dropped man fiercely waved

‘The girl waved fiercely to the man who dropped a recorder at a neighboring restaurant.’

14. 2. 女の子がビール瓶を近所のレストランで落とした男性に力いっぱい手をふった。

Girl-NOM beer bottle- ACC neighboring at a restaurant dropped man fiercely waved

‘The girl waved fiercely to the man who dropped a beer bottle at a neighboring restaurant.’

14. 3. 女の子が近所のレストランでリコーダーを落とした男性に力いっぱい手をふった。

Girl-NOM neighboring at a restaurant recorder- ACC dropped man fiercely waved

14. 4. 女の子が近所のレストランでビール瓶を落とした男性に力いっぱい手をふった。

Girl-NOM neighboring at a restaurant beer bottle- ACC dropped man fiercely waved

15. 1. 小学生が絵の具をテーブルで思いっきりこぼしたサラリーマンに大声で文句を言った。

Schoolchild-NOM paint -ACC on the table wildly spilled businessman loudly complained

‘The schoolchild complained loudly to the businessman who wildly spilled paint on the table.’

15. 2. 小学生がビール瓶をテーブルで思いっきりこぼしたサラリーマンに大声で文句を言った。

Schoolchild-NOM beer bottle-ACC on the table wildly spilled businessman loudly complained

‘The schoolchild complained loudly to the businessman who wildly spilled a beer bottle on the table.’

15. 3. 小学生がテーブルで思いっきり絵の具をこぼしたサラリーマンに大声で文句を言った。

Schoolchild-NOM on the table wildly paint -ACC spilled businessman loudly complained

15. 4. 小学生がテーブルで思いっきりビール瓶をこぼしたサラリーマンに大声で文句を言った。

Schoolchild-NOM on the table wildly beer bottle-ACC spilled businessman loudly complained

16. 1. ちびっ子がプラモデルを手に大切そうに持っている女優に礼儀正しくサインを求めた。

Child-NOM plastic model-ACC in hand carefully had actress politely an autograph asked

‘The child politely asked for an autograph to the actress who had a plastic model carefully in her hand.’

16. 2. ちびっ子がカクテルを手に大切そうに持っている女優に礼儀正しくサインを求めた。

Child-NOM cocktail-ACC in hands carefully had actress politely an autograph asked

‘The child politely asked for an autograph to the actress who had a cocktail carefully in her hand.’

16. 3. ちびっ子が手に大切そうにプラモデルを持っている女優に礼儀正しくサインを求めた。

Child-NOM in hand carefully plastic model-ACC had actress politely an autograph asked

16. 4. ちびっ子が手に大切そうにカクテルを持っている女優に礼儀正しくサインを求めた。

Child-NOM in hands carefully cocktail-ACC had actress politely an autograph asked

17. 1. 幼稚園児が積み木を手にさりげなく持っている会社員をトコトコと追いかけた。

Kindergartner-NOM building blocks-ACC in hands casually had businessman slowly followed

‘The kindergartner followed slowly the businessman who casually had building blocks in his hands.’

17. 2. 幼稚園児が名刺を手にさりげなく持っている会社員をトコトコと追いかけた。

Kindergartner-NOM name card-ACC in hands casually had businessman slowly followed

‘The kindergartner followed slowly the businessman who casually had a name card in his hands.’

17. 3. 幼稚園児が手にさりげなく積み木を持っている会社員をトコトコと追いかけた。

Kindergartner-NOM in hands casually building blocks-ACC had businessman slowly followed

17. 4. 幼稚園児が手にさりげなく名刺を持っている会社員をトコトコと追いかけた。

Kindergartner-NOM in hands casually name card-ACC had businessman slowly followed

18. 1. 園児が三輪車を近所の公園で運んでいる先生に力いっぱい手をふった。

Kindergartner-NOM tricycle-ACC in a nearby park carrying teacher fiercely waved

‘The kindergartner waved fiercely to the teacher who was carrying a tricycle in a nearby park.’

18. 2. 園児がオートバイを近所の公園で運んでいる先生に力いっぱい手をふった。

Kindergartner-NOM motorcycle-ACC in a nearby park carrying teacher fiercely waved

‘The kindergartner waved fiercely to the teacher who was carrying a motorcycle in a nearby park.’

18. 3. 園児が近所の公園で三輪車を運んでいる先生に力いっぱい手をふった。

Kindergartner-NOM in a nearby park tricycle-ACC carrying teacher fiercely waved

18. 4. 園児が近所の公園でオートバイを運んでいる先生に力いっぱい手をふった。

Kindergartner-NOM in a nearby park motorcycle-ACC carrying teacher fiercely waved

19. 1. 女優がドレスを近所でしぶしぶ購入したおじいさんに詳しく話をきいた。

Actress-NOM dress-ACC in the neighborhood reluctantly bought old man closely listened to a story

‘The actress listened closely to a story from the old man who reluctantly bought a dress in the neighborhood.’

19. 2. 女優が腹巻きを近所でしぶしぶ購入したおじいさんに詳しく話をきいた。

Actress-NOM belly warmer-ACC in the neighborhood reluctantly bought old man closely listened to a story

‘The actress listened closely to a story from the old man who reluctantly bought a belly warmer in the neighborhood.’

19. 3. 女優が近所でしぶしぶドレスを購入したおじいさんに詳しく話をきいた。

Actress-NOM in the neighborhood reluctantly dress-ACC bought old man closely listened to a story

19. 4. 女優が近所でしぶしぶ腹巻きを購入したおじいさんに詳しく話をきいた。

Actress-NOM in the neighborhood reluctantly belly warmer-ACC bought old man closely listened to a story

20. 1. おばあさんが扇子をバッグの中に密かに持っている女子高生に優しく質問した。

Old lady-NOM folding fan-ACC in a bag secretly had high school girl gently asked a question

‘The old lady gently asked a question to the high school girl who secretly had a folding fan in a bag.’

20. 2. おばあさんがマスカラをバッグの中に密かに持っている女子高生に優しく質問した。

Old lady-NOM mascara-ACC in a bag secretly had high school girl gently asked a question

‘The old lady gently asked a question to the high school girl who secretly had mascara in a bag.’

20. 3. おばあさんがバッグの中に密かに扇子を持っている女子高生に優しく質問した。

Old lady-NOM in a bag secretly folding fan-ACC had high school girl gently asked a question

20. 4. おばあさんがバッグの中に密かにマスカラを持っている女子高生に優しく質問した。

Old lady-NOM in a bag secretly mascara-ACC had high school girl gently asked a question

21. 1. 化学者が講演を教室で楽しそうにしている中学生に厳しくアドバイスした。

Chemist-NOM lecture-ACC in the classroom happily doing junior high school student strictly advised

‘The chemist advised strictly the junior high school student who was happily giving a lecture in the classroom.’

21. 2. 化学者が宿題を教室で楽しそうにしている中学生に厳しくアドバイスした。

Chemist-NOM homework-ACC in the classroom happily doing junior high school student strictly advised

‘The chemist advised strictly the junior high school student who was happily doing homework in the classroom.’

21. 3. 化学者が教室で楽しそうに講演をしている中学生に厳しくアドバイスした。

Chemist-NOM in the classroom happily lecture-ACC doing junior high school student strictly advised

21. 4. 化学者が教室で楽しそうに宿題をしている中学生に厳しくアドバイスした。

Chemist-NOM in the classroom happily homework-ACC doing junior high school student strictly advised

22. 1. 小学生がクレヨンをポケットの中にこっそり持っているビジネスマンをじっと見つめた。

Schoolchild-NOM crayon-ACC in the pocket secretly carrying businessman fixedly stared

‘The schoolchild stared fixedly at the businessman was secretly carrying a crayon in his pocket.’

22. 2. 小学生がひげ剃りをポケットの中にこっそり持っているビジネスマンをじっと見つめた。

Schoolchild-NOM shaver-ACC in the pocket secretly carrying businessman fixedly stared

‘The schoolchild stared fixedly at the businessman was secretly carrying a shaver in his pocket.’

22. 3. 小学生がポケットの中にこっそりクレヨンを持っているビジネスマンをじっと見つめた。

Schoolchild-NOM in the pocket secretly crayon-ACC carrying businessman fixedly stared

22. 4. 小学生がポケットの中にこっそりひげ剃りを持っているビジネスマンをじっと見つめた。

Schoolchild-NOM in the pocket secretly shaver-ACC carrying businessman fixedly stared

23. 1. 病人が点滴をひとりで退屈そうにしている大学生にこっそりと近づいた。

Sick person-NOM IV drip-ACC alone boringly doing university student secretly approached

‘The sick person secretly approached the university student who was getting an IV drip alone boringly.’

23. 2. 病人が筋トレをひとりで退屈そうにしている大学生にこっそりと近づいた。

Sick person-NOM exercise-ACC alone boringly doing university student secretly approached

‘The sick person secretly approached the university student who was exercising alone boringly.’

23. 3. 病人がひとりで退屈そうに点滴をしている大学生にこっそりと近づいた。

Sick person-NOM alone boringly IV drip-ACC doing university student secretly approached

23. 4. 病人がひとりで退屈そうに筋トレをしている大学生にこっそりと近づいた。

Sick person-NOM alone boringly exercise-ACC doing university student secretly approached

24. 1. 教授が論文を机で忙しそうに書いている小学生を大げさに褒めたたえた。

Professor-NOM academic paper-ACC on desk busily writing schoolchild exaggeratedly complimented

‘The professor complimented exaggeratedly the schoolchild who was busily writing an academic paper on a desk.’

24. 2. 教授が絵日記を机で忙しそうに書いている小学生を大げさに褒めたたえた。

Professor-NOM picture diary-ACC on desk busily writing schoolchild exaggeratedly complimented

‘The professor complimented exaggeratedly the schoolchild who was busily writing a picture diary on a desk.’

24. 3. 教授が机で忙しそうに論文を書いている小学生を大げさに褒めたたえた。

Professor-NOM on desk busily academic paper-ACC writing schoolchild exaggeratedly complimented

24. 4. 教授が机で忙しそうに絵日記を書いている小学生を大げさに褒めたたえた。

Professor-NOM on desk picture diary-ACC busily writing schoolchild exaggeratedly complimented
